# Efficacy of different remineralization agents on microhardness and chemical composition of enamel white spot lesion

**DOI:** 10.12688/f1000research.149166.2

**Published:** 2024-09-03

**Authors:** Rafal Ghanim Rahman, Ban Sahib Diab

**Affiliations:** 1 preventive and pedodontics, University of Baghdad, Baghdad, Baghdad Governorate, Iraq; 2 Prof. Department of Preventive and Pedodontic Dentistry, University of Baghdad, Baghdad, Iraq

**Keywords:** BAG, CPP-ACPF, Remineralization, white spot lesion.

## Abstract

**Background:**

White spot lesions (WSLs) are frequently linked with low microhardness and mineral content changes. several strategies have been employed to deal with these problems. This investigation aimed to analyze the microhardness and mineral content changes after remineralization with bioactive glass (BAG) and casein phospho-peptide-amorphous calcium phosphate with fluoride (CPP-ACPF).

**Methods:**

Twenty sound maxillary first premolars extracted were used to obtain a total of one hundred enamel samples. forty enamel slabs were split into four experimental groups (n = 10 each): Group I, BAG; Group II, BAG+CPP-ACPF; Group III, CPP-ACPF varnish; and Group IV, artificial saliva (negative control). To create artificial WSLs, all samples were preserved in a prepared demineralizing agent for 72 h before treatment with remineralizing agents. Vickers microhardness test was performed. Additionally, 60 enamel samples were selected for analysis using energy dispersive spectroscopy (EDX) and assigned to six experimental groups; the first four groups were similar to that used in the microhardness test along with Group V: WSLs, and Group VI: baseline. The statistical analyses employed in this study included Tukey’s HSD (p<0.05), one-way ANOVA, and Shapiro-Wilk.

**Result:**

Regarding surface microhardness, the BAG+CPP-ACPF group showed the most favorable recovery, which was better than the outcomes of the BAG and CPP-ACPF groups. A statistically significant change (p <0.05) was not observed between them. Similarly, for mineral content change, the BAG+CPP-ACPF group demonstrated the greatest result, The BAG group came next, and the CPP-ACPF group came last.

**Conclusion:**

The BAG+CPP-ACPF group might be regarded as the best course of treatment for enhancing both the surface microhardness and mineral content (Ca, P), while the control group (Artificial saliva) showed the least satisfactory results in comparison. After demineralization, mineral content and microhardness decreased in all samples. Therefore, BAG+CPP-ACPF significantly improved the surface microhardness and mineral content.

## Introduction

In dentistry, an early demineralization of enamel, either on the surface or slightly below it, is mentioned as a “white spot lesion” (WSL), This is dental caries’ first obvious sign. These lesions’ reversibility suggests that substantial dental procedures may not be necessary to restore the tooth structure. WSLs must be addressed right away, though, since their surfaces may become porous and lose their original gloss, which raises issues regarding their aesthetics and their development. ^1^ WSL is reversible in its early stages if remineralization mechanisms are initiated. ^2^ Up to 96% of orthodontic patients have WSLs owing to the difficulty in cleaning teeth when attachments are present, leading to food retention and plaque accumulation. ^3^ During subsurface demineralization, the enamel surface layer normally remains intact; nevertheless, if treatment is not received, it will eventually decompose into a real hollow. ^4^ Several conservative techniques were developed to treat WSLs. fluoride (F) is thought to be the primary factor influencing remineralization. Fluorides can be applied in dentistry practices in a variety of ways, including toothpaste, gels, rinses, and varnishes. Of all these types that are accessible, varnish yields the best effects. Fluoride varnish increases fluoride concentration on the tooth surface by acting as a reservoir for fluoride. Recently, the application of substances like casein phospho-peptide-amorphous calcium phosphate (CPP-ACP) has been explored as a substitute for fluoride for promoting remineralization. ^5^ CPPs are peptides formed from the casein milk protein. ACP is a form of calcium and phosphate, which is maintained by CPP. ^6^^,^^7^ It may release fluoride, phosphate, and calcium in response to acidogenic challenges, slows demineralization, and promotes remineralization. It attaches very easily to soft tissues, plaque, saliva pellicle, and even the hydroxyapatite portion of enamel. ^8^ One of the non-invasive techniques used to treat white spot lesions is preconditioning with air abrasion, which increases surface porosity and allows mineral ions to deposit into the lesion’s underlying body, improving the effectiveness of remineralizing agents by causing the lesion surface to become active. ^9^ Owing to its unique qualities like remineralization capacity, bioactive glass 45S5 (BAG) was manufactured. ^10^^,^^11^ WSLs were remineralized using gel and paste forms of BAG. ^12^ To promote WSL remineralization, the surface was prepared using air abrasion, and then the application of bioactive glass. However, this method is time-consuming. ^13^ BAG is a therapeutic powder made entirely of a bioactive glass called Novamin that reacts quickly on the surface to produce hydroxycarbonate apatite (HCA) in the presence of water. HCA and natural tooth minerals are chemically similar. ^14^ Once set by eating and brushing the teeth, the majority of fluoride varnishes put on the enamel surface are removed easily. Retention on the tooth surface is essential for materials that successfully encourage remineralization, precondition the teeth surface by air abrasion with BAG, and then apply varnish consisting of fluoride, calcium, and phosphate to increase the efficacy of remineralization. It is a novel approach aimed at assessing the alteration in enamel following preconditioning the surface with BAG + CPP-ACPF. The null hypotheses state that there was no difference in enamel microhardness and mineral content between the experimental and control groups While the alternative hypothesis states there was a difference in enamel microhardness and mineral content between the experimental and control groups. This study analyze the efficacy of BAG air abrasion preconditioning and CPP-ACPF varnish remineralization and contrast its effectiveness with BAG alone and CPP-ACPF varnish alone. Microhardness and energy dispersive spectroscopy (EDX) were used for analysis.

## Methods

### Samples selection

This is an *in-vitro* study, twenty sound permanent maxillary first premolars were obtained from patients aged 12–20 years who underwent orthodontic treatment relying on a health research committee College of Dentistry University of Baghdad approved ethical protocol (Ref No. 724) dated 28/12/2022. The samples were cleaned with Tap water and polished using non-fluoridated slurry using prophylactic brushes attached to a slow-speed handpiece. The samples were then stored in a thymol solution (DR Thym™, InnuScience, Canada) of 0.1% (anti-microbial solution to restrict bacterial proliferation) for 1 week. Before use, it was kept in distilled water in a refrigerator at a temperature of 4 ± 0.1 °C until further use. ^15^

### Preparation of enamel sample

Ten teeth were chosen for the study on EDX, and ten samples for the investigation on surface microhardness. Excluded from the study were teeth with cracks, stains, fluorosis, or developmental abnormalities (assessed using a light curing unit and a magnifying lens from (Fukai, China). The samples had comparable dimensions by measuring the mesiodistal (M-D) lengths using a digital Vernier (figure S3). Sections of the teeth were obtained to expose the lingual and buccal surfaces. ^16^

Estimation of Sample size by Utilizing G power 3.1.9.7 (via Franz-Faul, Universidad Kiel program, Germany) with the power of study 80%, alpha error of probability 0.05, the correlation between time points is 0.5 and the effect size of F is 0.25 with four groups and three-time points, with all these conditions, the sample size is 36 samples, thus 40 samples for microhardness were enough for this study while 60 samples for EDX analysis. Ten teeth were selected for the microhardness test, and four sections of enamel slabs were obtained from each tooth using a diamond disc bur for root removal and a large amount of water to prevent damage to the enamel. Enamel slabs were obtained using an XP precision sectioning saw (Pelco, USA). Their measurements were checked using a digital caliper **(**to get precise measurements (approximately 3 mm width × 3 mm length and 1.5 mm thick). ^17^ The samples were embedded in acrylic resin using a silicone mold that had the following dimensions: 0.5 cm × 1 cm × 1 cm. The resin was poured into the mold (Figure-S4A), and the enamel slab (Figure-S4B) was placed inside, with the outer enamel surface exposed **(Figure S4)**. For “EDX” another ten premolar teeth were used to obtain the elemental composition of the enamel and ultrastructural information. Six sections of enamel slabs, three each from the buccal and palatal surfaces (3mm length × 1.5 mm thickness and 1.5 mm width), were obtained from each tooth placed in resin molds. The samples EDX were obtained without polishing to protect the microstructure. ^18^ Silicon carbide paper of 1200 grit was used for 10 s for polishing microhardness samples (Laryee Technology CO. LTD, China) under water cooling to obtain smooth and flat surfaces, followed by ultrasonic cleaning for 4 min to remove surface debris. ^19^

### Sample grouping and study design

For the microhardness study:

**Group I (BAG):** In 10 sound enamel slabs with existing WSL, the surface is conditioned using BAG (Sylc ^®^ powder) (Denfotex Research Ltd, Inverkeithing, UK).

**Group II (BAG+CPP-ACPF):** In 10 sound enamel slabs with existing WSL, the surface is conditioned using BAG and then using CPP-ACPF varnish (MI varnish-GC company- Japan).

**Group III (CPP-ACPF):** Ten sound enamel slabs with existing WSL were treated using CPP-ACPF varnish

**Group IV (Artificial saliva):** Ten sound enamel slabs with existing WSL were kept in the saliva without any intervention.

For EDX examination, sixty enamel slabs were used, and six groups were randomly assigned to receive each specimen from a single tooth:

“Groups I to IV were similar to the groups in the microhardness study; additionally, the following two groups were included.”

**Group V (Baseline):** Ten sound enamel slabs.

**Group VI (WSLs):** Ten sound enamel slabs with existing WSL, without further intervention.

### Demineralization process

The samples were placed in a demineralized solution (Pioneer CO., Sulaymaniyah, Iraq) (acetic acid = 50 mM, NaCl = 100 mM, NaF = 1 ppm, NaN _3_ = 5 mM, NaH _2_PO _4_–2H _2_O = 10 mM, CaCl _2_-2H _2_O = 2.2 mM) to create an artificial white spot lesion. 1 M NaOH solution was used to adjust the pH to 4.5 r (SD Fujian, China) **(Figure S5A, Figure S5B)**. ^20^ For four days, the demineralization process was conducted at 37 °C. Following a 30-second rinse in deionized water, the samples were also preserved in water that had been distilled.

### Approach of remineralization

*A. Air abrasion with BAG group*

The BAG powder Sylc ^®^ (Denfotex Research Ltd, Inverkeithing, UK) **(Figure S2)** which is a calcium sodium phosphor-silicate material with particle size ranging from (25 to 120 μm) was introduced to the reservoir according to the manufacturer’s recommendations. With the air stream encased in a deionized water barrier, performing air abrasion for 10 seconds with a circular movement using an Aqua Care Air Abrasion & Polishing System from VELOPEX International operating at an air pressure of 80 psi and a feed rate of 1 **(Figure S6)**. The apparatus comprised a disposable plastic tip and a handpiece with a 0.8 mm diameter. Followed by washing with running water, which was stored in artificial saliva (20 mL per specimen) with a change of saliva every 24 hours for 14 days until final measurement. Artificial saliva (Pioneer CO., Sulaymaniyah, Iraq) contains (Potassium thiocyanate = 0.01; Sorbitol = 1, Potassium chloride = 1, Magnesium chloride = 0.05; Potassium phosphate = 0.04; Sodium carboxymethyl cellulose = 10, Sodium chloride = 1, Sodium fluoride = 0.0002,100 milliliters of boiling water and cooling, sodium carboxymethyl cellulose (985.5) mm of deionized water, was dissolved.

*B. Air abrasion with BAG and CPP-ACPF group*

After surface preconditioning with air abrasion as mentioned above, then applying CPP-ACPF varnish (MI varnish-GC company) to each sample **(Figure S1)**, a single unit dosage packet has 1 mL of MI and 0.55 g/0.5 mL. There are 50 mg of NaF (22.6 mg F ion) in one varnish. it was allowed to air-dry for 20 seconds to ensure that it was set and became dry. To ensure that a consistent amount of fluoride varnish was applied to each specimen, a single-dose form was used. Subsequently, the slabs were maintained in saliva for 14 days, with a replacement of saliva every 24 hours.

*C. CPP-ACPF group*

After applying CPP-ACPF to each sample, it was allowed to air-dry for 20 s to ensure that it was set and became dry. To ensure that a consistent amount of fluoride varnish was applied to each specimen, a single-dose form was used. Subsequently, the slabs were maintained in saliva for 14 days, with a replacement of saliva every 24 hours.

*D. Control group*

Samples from this group were stored in saliva without any intervention.

### Microhardness

Before the microhardness assessment, samples were kept in a moist environment. SMH was tested on each block surface. Vickers microhardness tester (Laryee, Model: HVS-1000, Beijing Time High Technology Ltd, China) was used to examine the hardness profile employing a 200-gf load and a pyramid-shaped indenter based on diamond squares for 15 s resulting in a diagonal shape on the surface (n=3 per sample) **(Figure S7)**. The Vickers hardness numbers (VHN) were obtained for each sample after three marks were made at the middle, upper, and lower ends of the enamel surface while leaving a reasonably sound region in between. ^21^

The recovery of enamel surface microhardness (% SMH) was calculated by Cury et al. ^22^

100 (SMH post-treatment − SMH caries)/baseline SMH − SMH caries

### Energy-dispersive X-ray spectrometry (EDX)

EDX (INSPECT F50, FEI Company, Eindhoven, The Netherlands) was used to determine the elemental compositions of the produced enamel samples (voltage: 20 kV; working distance: 10 mm; spatial resolution: 100 nm; count time: 60 s). ^12^ Three measurements were obtained for each sample and the mean values were calculated **(Figure S8)**. The weight of calcium and phosphorus contents was calculated. EDX was obtained at the baseline group for intact enamel before demineralization and after demineralization (WSL group), and finally obtained after the remineralization period for each group. ^23^

### Statistical analysis

Data description, analysis, and demonstration were performed using Statistical software for Social Science SPSS version 21.

Shapiro–Wilk test was used to determine whether the data were distributed normally. For group comparisons, repeated-measures analysis of variance (ANOVA) was employed. To compare subgroups, Tukey’s honest significant difference (HSD) test was used for post-hoc analysis. Statistical significance was set at P<0.05.

## Results

### Surface microhardness

Shapiro-Wilk test results for normality showed that surface Microhardness at (baseline, demineralization, and after remineralization) is normally distributed among groups as Table 1 indicates that there were no significant differences (p>0.05).

**Table 1.** Shapiro-Wilk Test for Normality test of Surface microhardness.

Phases	Groups	Shapiro-Wilk		
		Statistic	Df	P value
Baseline	BAG	0.908	10	0.266
	BAG+ CPP-ACPF	0.867	10	0.091
	CPP-ACPF	0.940	10	0.548
	Control	0.954	10	0.717
Demineralization	BAG	0.853	10	0.054
	BAG + CPP-ACPF	0.952	10	0.693
	CPP-ACPF	0.916	10	0.323
	Control	0.943	10	0.586
Remineralization	BAG	0.975	10	0.930
	BAG + CPP-ACPF	0.940	10	0.552
	CPP-ACPF	0.968	10	0.872
	Control	0.909	10	0.274

**Figure 1.**
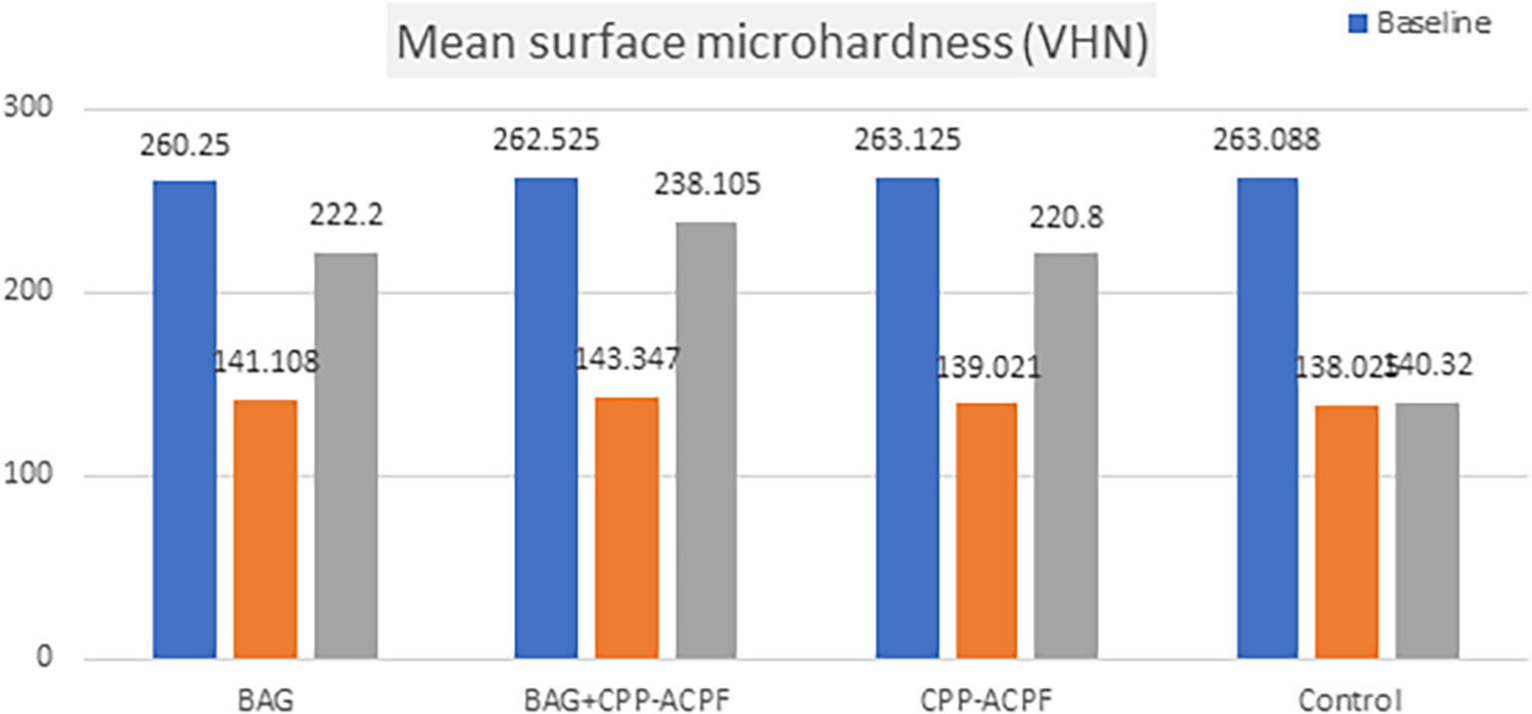
Bar chart demonstrating the differences in microhardness among groups and stages.

The surface microhardness mean value decreased at baseline and demineralization phase for each group and subsequently, it increased significantly at the remineralization phase. The greatest increase (larger % of recovery) from the demineralization to remineralization phase was in the BAG+CPP-ACPF group, followed by the BAG group and then the CPP-ACPF group, respectively, with little difference between these two groups, while the lowest was in the control group, as shown in Table 2 and Figure 1.

**Table 2.** Descriptive and statistical test of surface microhardness in (kg/mm ^2^) at three time periods among groups.

Groups		Baseline	Demin.	Remin.	F	P value	% Recovery
BAG	Min.	250.75	135.17	213.30	1623.205	0.000	68.128
	Max.	270.00	151.08	230.00			
	Mean	260.250	141.108	222.200			
	±SD	5.587	4.617	5.327			
BAG+ CPP-ACPF	Min.	248.50	137.67	225.25	2063.395	0.000	79.495
	Max.	271.00	148.80	248.80			
	Mean	262.525	143.347	238.105			
	±SD	7.199	3.994	8.089			
CPP-ACPF	Min.	257.13	131.00	210.00	1605.968	0.000	66.085
	Max.	272.25	147.00	231.00			
	Mean	263.125	139.021	220.800			
	±SD	5.243	5.582	6.763			
Control	Min.	253.50	132.08	130.67	1508.906	0.000	1.647
	Max.	271.63	148.00	145.83			
	Mean	263.088	138.025	140.320			
	±SD	4.706	5.136	5.029			
F		0.557	2.352	471.083			
P value		0.647	0.088	0.000			

ANOVA showed no significant differences in the surface microhardness values among all groups at the baseline and demineralization phases (P>0.05), although significant statistical differences were noted between the groups (P difference among the four groups in the remineralization phase).

### Effect of phases in each group

Table 3 displays multiple pairwise comparisons of Microhardness by groupings across phases using the Tukey Honestly Significant Difference (Tukey HSD) test. Except for the control group (p>0.05), all groups had a greater mean difference in the remineralization phase as compared to the demineralization phase (p<0.05). All groups’ mean differences during the baseline phase, however, were greater than those at the remineralization phase (p<0.05).

**Table 3.** Tukey HSD test for Microhardness comparison between phases in each group.

GROUPS	Phases		Mean Difference	P value
BAG	Baseline	Demin.	119.142	0.000
		Remin.	38.050	0.000
	Demin.	Remin.	-81.092	0.000
BAG+ CPP-ACPF	Baseline	Demin.	119.178	0.000
		Remin.	24.420	0.000
	Demin.	Remin.	-94.758	0.000
CPP-ACPF	Baseline	Demin.	124.104	0.000
		Remin.	42.325	0.000
	Demin.	Remin.	-81.779	0.000
Control	Baseline	Demin.	125.063	0.000
		Remin.	122.768	0.000
	Demin.	Remin.	-2.295	0.944

MD = mean difference.

### EDX assessment among groups

**Figure 2.**
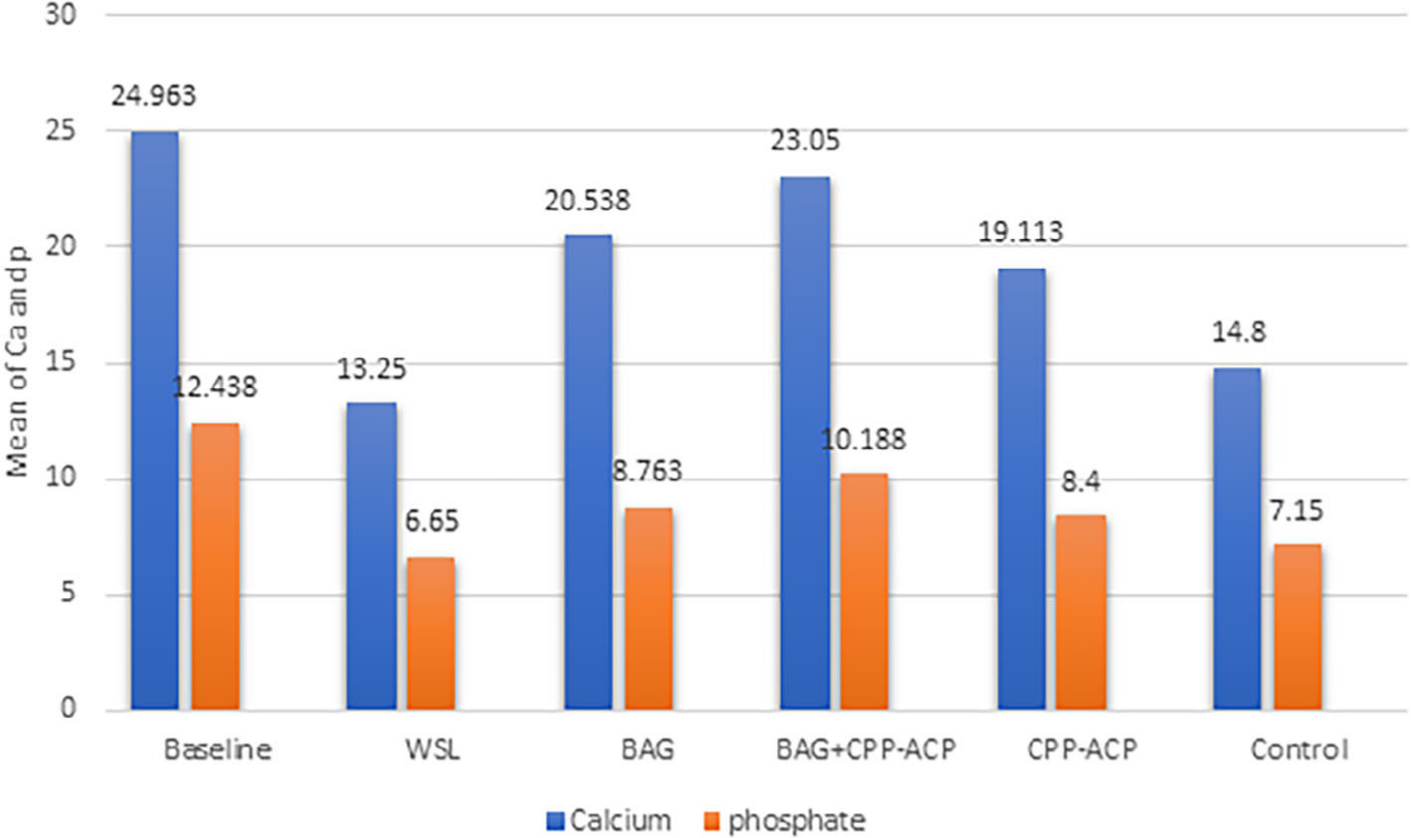
Bar graph showing Calcium and phosphorus content analysis of the enamel surface with EDX measurement.

The information from the EDX assessment for all groups about the weight percentages of phosphorous and calcium is shown in Figure 2. The weight percentage of both calcium and phosphorus decreased after demineralization. An increase in the weight percentage for Ca and P after remineralization in the BAG and CPP-ACPF groups compared with the WSL group, and the lowest was observed in the demineralization group (WSL), with significant differences among groups. The maximum values of Ca and P were recorded in the BAG+CPP-ACPF group. Minimal increase was noticed in the weight percentage of Ca, and P for groups treated using artificial saliva (the control group) as compared with the demineralization stage.

## Discussion

It is crucial to take the most suitable course of action to protect tooth enamel and increase dental caries resistance; however, this can be difficult because of the intricate structural and functional interactions. This study investigated enamel remineralization using BAG air abrasion and CPP-ACP, and its effect on mineral content and microhardness. Basic chemical models have been utilized to produce artificial lesions in most in vitro cariology investigations. ^24^ It offers clear benefits, such as easy research, time, controlled experimental conditions, and repeatability of the results. Because natural WSLs on enamel vary greatly in size, shape, and mineral content, natural WSL teeth were replaced using artificial WSLs in human premolars. ^25^ To create artificial enamel lesions for cariology research, these lesions should be considered acceptable. Changes in mechanical properties typically coincide with a shift in the chemical makeup of demineralized enamel. Surface microhardness measurement is regarded as an accurate and effective tool for assessing tooth surface modification following the de- and re-mineralization processes. ^26^ VHN obtained during the baseline microhardness measurements satisfied the typical enamel tissue VHN range of 248.5–272.25 which coincides with the results of Meredith et al. ^27^ Following demineralization, the microhardness of each group of enamel samples decreased from 131 to 151.08. Within the constraints of this research, the findings showed that hardness tended to rise and then regain in the BAG+CPP-ACPF group, followed by BAG then by CPP-ACPF alone with a significant difference.

The control group showed the smallest difference. The results indicated significantly higher microhardness in the BAG group than that in the CPP-ACPF group. This coincides with an earlier in vitro study by Alessa N, Shah SA et al. ^28^ that compared several remineralizing agents, and found that BAG is a more effective agent in treating initial caries. Furthermore, Mehta et al. ^29^ discovered that Novamin remineralized the carious lesion more successfully than CPP-ACP, which may have been due to the bioactivity of BAG in the formation of hydroxycarbonate apatite (HCA) layer from its reactions with tissue fluids. Bioglasses that come in contact with bodily fluids cause diverse hydrated silicate species, as well as Phosphate, calcium, and sodium, to dissolve and leach quickly from the glass surface. A poly-condensed silica-rich gel layer was developed on the glass surface, offering calcium phosphate precipitation nucleation sites, the reduced CPP-ACPF hardness values when compared with BAG may be ascribed to its amorphous structure, which prevents it from adhering to the tooth enamel surface, as BAG does. Consequently, it does not remineralize the tooth surface over an extended period to increase the hardness. ^30^ Dong et al. ^31^ examined several types of BAG, one of which included 45S5, and demonstrated a mineralized layer with a 4 μm average thickness had formed, filling enamel porosities. The results of this research coincide with those of the study by Chabuk and Al-Shamma ^32^ which concluded that BAG improved enamel hardness. The effect of CPP-ACPF on enamel hardness was much greater than that in the artificial saliva group). This finding coincides with Attar IJ, Ghaib NH, ^33^ Al-Janabi, S.Z.; Al-Dahan, ^34^ and Bandekar et al. ^35^ demonstrating the effectiveness of CPP-ACPF in remineralizing artificial carious lesions. Reynolds et al. ^36^ concluded that this is connected to the capacity of CPP to function as a bioavailable calcium and phosphate using ACP localization on the tooth’s surface and assisting in the preservation of a supersaturated state of the tooth enamel, whereby de-mineralization is prevented and remineralization is improved. Contrary to our findings, an in vitro investigation by Vyavhare et al. ^37^ discovered that CPP-ACP does not exhibit a significant amount of surface remineralization. This discrepancy may have resulted from differences in experimental design and treatment regimens employed.

The weight percentages of calcium and phosphorus ions were used to assess mineral alterations on the enamel surface quantitatively. The results of EDX discovered that calcium and phosphorus declined after demineralization, referring to rapid loss of minerals, and rising following remineralization in all groups, showing a mineral gain. The BAG+CPP-ACPF group had the highest mean calcium and phosphorus levels, followed by the BAG and CPP-ACPF groups, whereas the control and WSL groups had the lowest mean levels, with a significant difference between the groups. The average calcium and phosphorus levels were substantially higher in the BAG group than in the CPP-ACPF group. An in vitro method used in research by Narayana et al. ^38^ revealed that bioactive glass had more efficacy in remineralization than CPP-ACPF. This explains that phosphate and calcium ions were continuously released into the surrounding environment for several days, acting as reservoirs for these ions. Burwell et al. ^39^ concluded that the BAG released ions and changed into HCA for a maximum of 14 days, which was deficient in CPP-ACPF action. Moreover, the CPP-ACPF group displayed higher mean calcium and phosphorus values than the control group. This could be attributed to the capacity of the CPP-ACP complex to act as a transporter, carrying fluoride, calcium, and phosphorus ions to the tooth surface. Somasundaram et al. ^40^ concluded that by depressing de-mineralization and promoting remineralization, the CPP-ACPF preserves the mineral saturation levels, especially those of phosphate and calcium, at the tooth surface. Because CPP-ACPF can remineralize surface lesions but not early enamel caries at the subsurface level, it can prevent fast calcium and phosphate precipitation, which lowers the calcium and phosphorus ratios. This is why the results of this research showed that CPP-ACPFF had lower calcium and phosphorus percentages than BAG. To the best of our knowledge, no investigations have been performed in the BAG+CPP-ACPF-treated group employing BAG to treat WSL before applying CPP-ACPF. The combined effect of BAG+CPP-ACPF significantly improved the surface microhardness and mineral content (calcium and phosphorus). The BAG rich in calcium, phosphate, and silica was gradually substituted by hydrogen ions, and CPP-ACPF acted as a transporter for calcium and phosphate to enhance remineralization of the enamel surface, providing a synergistic effect on the microhardness and mineral content of enamel and explain the results of this investigation. Based on the outcomes derived from the current study, the null hypothesis is thus rejected. This is due to a clear and noteworthy variation in surface microhardness and mineral content observed across the various stages (baseline, demineralization, and remineralization), as well as within the three methodologies (BAG+CPP-ACPF, BAG, and CPP-ACPF).

This is an in vitro study, it is difficult to create an accurate and identical environment typical of the oral cavity with its multifactorial influences such as enzymes, plaque, salivary proteins, and continuous salivary flow, all of which have a substantial impact on the effectiveness of the materials employed and, eventually, the outcomes that are achieved. The intricacy and traits of enamel lesions might not be completely captured by the use of artificial lesions. The Sample size and duration of the study may affect statistical results. Further investigation is required to generalize the results of this research. A perfect proportion between the overall surface quality and hardness can also be maintained by assessing the efficacy of various polishing methods and investigating the use of surface modifiers.

## Conclusion

Considering these results, it can be stated that the BAG+CPP-ACPF group demonstrated potential utility in increasing WSL remineralization, as evidenced by its ability to enhance enamel remineralization much better than the commercially available BAG and CPP-ACP groups. However, its clinical utility needs to be confirmed by additional in vivo testing.

### Ethics and consent

This research project was approved by the research ethics committee of the College of Dentistry, University of Baghdad, Baghdad, Iraq. Ref. number: 724. Date: 28-12-2022. Written informed consent was obtained.

### Supplementary Figures

**Figure S1:** (MI varnish) Casein PhosphoPeptide-Amorphous Calcium Phosphate (CPP-APCF)

**Figure S2**: Bioactive glass 45S5 Sylc ^®^ powder

**Figure S3:** Measuring the dimensions of the tooth

**Figure S4: A:** silicon mold

**B:** Enamel slab in an acrylic block

**Figure S5: A:** Enamel slabs before demineralization

**B:** Enamel slabs with Artificial WSL after demineralization procedure.

**Figure S6:** AquaCare air abrasion device

**Figure S7:** Vickers microhardness tester

**Figure S8:** EDX device

## Data Availability

Figshare: Efficacy of different remineralization agents on microhardness and chemical composition of enamel white spot lesion. This project contains the following underlying data:
I. Raw data for microhardness test: https://doi.org/10.6084/m9.figshare.25638120.v1. ^41^ This project contains the following underlying data: surface Microhardness at three time periods for each remineralizing agent: Baseline, after demineralization, and after remineralization.
II. Raw data EDX results: https://doi.org/10.6084/m9.figshare.25669062.v1. ^42^ This project contains the following underlying data: surface mineral content (Ca, P) at the Baseline, after demineralization, and after remineralization for all groups. Data are available under the terms of the Creative Commons Attribution 4.0 International license (CC-BY 4.0). Pictures of the material and equipment used in the study, https://doi.org/10.6084/m9.figshare.25638144.v1. ^43^ Data are available under the terms of the Creative Commons Attribution 4.0 International license (CC-BY 4.0).
