# Supplementary material for: Efficacy of different remineralization agents on microhardness and chemical composition of enamel white spot lesion
Source: F1000Res. 2024 Sep 3;13:505. Originally published 2024 May 17. [Version 2] doi: 10.12688/f1000research.149166.2 (PMC11333879; doi:10.12688/f1000research.149166.2)
Supplement: Peer review history

# Reviewer report: Reviewer response for version 2

**DOI:** 10.5256/f1000research.170911.r349219

**Published:** 2024-12-24

**Authors:** Shereen Hafez Ibrahim

**Affiliations:** 1 Cairo University Faculty of Dentistry (Ringgold ID: 110152), Cairo, Cairo Governorate, Egypt

Manuscript title: Efficacy of different remineralization agents on microhardness and chemical composition of enamel white spot lesion

 The manuscript is well designed and clearly described. I would like to provide feedback to enhance its quality.

 1. Introduction and Background

 The introduction could benefit from a more concise explanation of the specific clinical challenges associated with WSL.

 White spot lesion is an important issue. Therefore, it is worth search for appropriate management to prevent further WSL progression and enhance remineralization of enamel.  Several laboratory and clinical studies have assessed the efficacy of different remineralization agents However, the author did not explain this laboratory study's novelty throughout the manuscript as a whole.

 The introduction should more clearly state the research gap that this study is addressing.

 Different remineralization approaches either classical or non-classical pathway need to be discussed in detail.

 In the methodology section, the authors measure the surface microhardness and elemental analysis EDX.

 Microhardness tests assess only surface; however, subsurface lesion was not assessed. thus, my point of view to carry quantitative assessment for effective remineralization of subsurface lesion are mandatory.

 The oral environment is extremely challenging to in laboratory study it is important to simulate the clinical condition by exposing the specimens to acidic challenges with thermal fluctuation after the suggested treatment protocol to accurately assess its efficacy.

 In the discussion section: This section would benefit from a more critical analysis of the limitations, comparing you results with previous studies.

 The conclusion could better reflect the study’s limitations.

Is the work clearly and accurately presented and does it cite the current literature?

Partly

If applicable, is the statistical analysis and its interpretation appropriate?

Yes

Are all the source data underlying the results available to ensure full reproducibility?

Yes

Is the study design appropriate and is the work technically sound?

Yes

Are the conclusions drawn adequately supported by the results?

Partly

Are sufficient details of methods and analysis provided to allow replication by others?

Yes

Reviewer Expertise:

i am professor of Conservative Dentistry, Cairo University, Egypt. I am an expert in the field of Nanotechnology and digital dentistry, and I am an editor of Scopus indexed textbook entitled “Nanotechnology in conservative dentistry” by Elsevier that has great prominent percentile on Scival.ORCID: 0000-0002-1047-124X

I confirm that I have read this submission and believe that I have an appropriate level of expertise to confirm that it is of an acceptable scientific standard, however I have significant reservations, as outlined above.

# Reviewer report: Reviewer response for version 2

**DOI:** 10.5256/f1000research.170911.r320290

**Published:** 2024-09-09

**Authors:** Rehab Samir Salma

**Affiliations:** 1 College of Dentistry El Alamein, AAST, New Alamein, Egypt

No further comments

 Thank you

Is the work clearly and accurately presented and does it cite the current literature?

Yes

If applicable, is the statistical analysis and its interpretation appropriate?

Yes

Are all the source data underlying the results available to ensure full reproducibility?

Yes

Is the study design appropriate and is the work technically sound?

Yes

Are the conclusions drawn adequately supported by the results?

Yes

Are sufficient details of methods and analysis provided to allow replication by others?

Yes

Reviewer Expertise:

Pediatric dentistry

I confirm that I have read this submission and believe that I have an appropriate level of expertise to confirm that it is of an acceptable scientific standard.

# Reviewer report: Reviewer response for version 1

**DOI:** 10.5256/f1000research.163586.r311070

**Published:** 2024-08-19

**Authors:** Shahzad Ali Shah

**Affiliations:** 1 Conservative dental sciences, College of dentistry, Qassim University, Qassim, Saudi Arabia

Based on the information provided,

 1.  the study was well designed and executed.

 2. The sample size mentioned was 20 which was divided into two groups. please explain, how the sample size was calculated, and what was the reason for extraction of these premolars?

 3. the references should be updated according to the guidelines for authors stated in the journal website.

 4. Some of the references are too old and should be replaced with new ones, here are some suggestions,

 Shah SA et al, 2024 [Ref 1]

 Alessa N et al, 2024 [Ref 2]

Is the work clearly and accurately presented and does it cite the current literature?

Yes

If applicable, is the statistical analysis and its interpretation appropriate?

I cannot comment. A qualified statistician is required.

Are all the source data underlying the results available to ensure full reproducibility?

Yes

Is the study design appropriate and is the work technically sound?

Yes

Are the conclusions drawn adequately supported by the results?

Yes

Are sufficient details of methods and analysis provided to allow replication by others?

Partly

Reviewer Expertise:

consultant restorative dentistry, Associate professor, Diploma in Medical education

I confirm that I have read this submission and believe that I have an appropriate level of expertise to confirm that it is of an acceptable scientific standard.

## References

[1] : Evaluation of Remineralizing Capacity of Tricalcium Phosphate, Nano-Hydroxyapatite and Ozone Remineralizing Agents on the Artificial Carious Lesion. *Indian J Dent Res* .2024;35(1) : 10.4103/ijdr.ijdr_704_22 84-87 10.4103/ijdr.ijdr_704_22 38934756

[2] : Assessment of Efficiency of Bioactive Glass, Self-Assembling Peptide, and Ozone Remineralising Agents on Artificial Carious Lesion. *Pesquisa Brasileira em Odontopediatria e Clínica Integrada* .2024;24: 10.1590/pboci.2024.018 10.1590/pboci.2024.018

# Response

**Published:** 2024-08-23

**Authors:** Rafal Ghanem

I would like to thank you for your comment and for reviewing.

 The reason for teeth extraction is for orthodontic purposes and I mentioned that in the text.

 I add a sample size estimation. 

 I replace the old reference with one of your suggestions.

 Thank you again for your worthy comment.

# Reviewer report: Reviewer response for version 1

**DOI:** 10.5256/f1000research.163586.r295143

**Published:** 2024-07-15

**Authors:** Rehab Samir Salma

**Affiliations:** 1 College of Dentistry El Alamein, AAST, New Alamein, Egypt

Dear authors, first, thank you for this interesting topic. In general, the manuscript is well written.

 However, there are few points to be addressed. 

 Introduction: You should include a null hypothesis for the study.

 Materials and methods: you should mention the type of this study. You didn’t mention sample size estimation. May be if the number of samples per group increased, this would have yield different results!! This could be confirmed if you did a sample size estimation. Please justify. There is lack of randomization. Results: Tables footnotes should be added.

Is the work clearly and accurately presented and does it cite the current literature?

Yes

If applicable, is the statistical analysis and its interpretation appropriate?

Yes

Are all the source data underlying the results available to ensure full reproducibility?

Yes

Is the study design appropriate and is the work technically sound?

Yes

Are the conclusions drawn adequately supported by the results?

Yes

Are sufficient details of methods and analysis provided to allow replication by others?

Yes

Reviewer Expertise:

Pediatric dentistry

I confirm that I have read this submission and believe that I have an appropriate level of expertise to confirm that it is of an acceptable scientific standard.

# Response

**Published:** 2024-08-23

**Authors:** Rafal Ghanem

Dear Dr. Rehab Samir Salma

 I would first like to thank you for reviewing my manuscript.

 I am very grateful for your comments.

 This is an in-vitro study.

 The null hypotheses state: There was no difference in enamel microhardness and mineral content between the experimental and control groups. 

 While the alternative hypothesis states there was a difference in enamel microhardness and mineral content between the experimental and control groups 

 Based on the outcomes derived from the current study, the null hypothesis is thus rejected. This is due to a clear and noteworthy variation in surface microhardness and mineral content observed across the various stages (baseline, demineralization, and remineralization), as well as within the three methodologies (BAG+CPP-ACPF, BAG, and CPP-ACPF).

 Sample size:

 Utilizing G power 3.1.9.7 (via Franz-Faul, Universidad Kiel program, Germany) with the power of study 80%, alpha error of probability 0.05, the correlation between time points is 0.5 and the effect size of F is 0.25 with four groups and three-time points, with all these conditions, the sample size is 36 samples, thus 40 samples for microhardness were enough for this study while 60 samples for EDX analysis

 Randomization:  The distribution of enamel slabs for microhardness and EDX groups in a random manner. 

 Each slab from the same tooth was distributed among the different groups in a random manner.
